# Trends in Scientific Reports on Cartilage Bioprinting: Scoping Review

**DOI:** 10.2196/15017

**Published:** 2019-08-28

**Authors:** Àngels Salvador Vergés, Meltem Yildirim, Bertran Salvador, Francesc Garcia Cuyas

**Affiliations:** 1 Digital Care Research Group Universitat de Vic - Universitat Central de Catalunya Barcelona Spain; 2 Research Group on Methodology, Methods, Models and Outcomes of Health and Social Sciences Department of Nursing, Faculty of Health Sciences and Welfare Universitat de Vic - Universitat Central de Catalunya Barcelona Spain; 3 Communication Department University Pompeu Fabra Barcelona Spain; 4 Catalan Society of Digital Health Hospital Sant Joan de Déu Universitat de Vic - Universitat Central de Catalunya Barcelona Spain

**Keywords:** cartilage 3D printing, knowledge, tissue engineering, surgery, cartilage repair, chondrogenesis, cartilage biomaterials

## Abstract

**Background:**

Satisfactory therapeutic strategies for cartilaginous lesion repair do not yet exist. This creates a challenge for surgeons and biomedical engineers and leads them to investigate the role of bioprinting and tissue engineering as viable treatments through orthopedic surgery, plastic surgery, and otorhinolaryngology. Recent increases in related scientific literature suggest that bioprinted cartilage may develop into a viable solution.

**Objective:**

The objectives of this review were to (1) synthesize the scientific advances published to date, (2) identify unresolved technical problems regarding human application, and (3) identify more effective ways for the scientific community to transfer their findings to clinicians.

**Methods:**

This scoping review considered articles published between 2009 and 2019 that were identified through searching PubMed, Scopus, Web of Science, and Google Scholar. Arksey and O'Malley’s five-step framework was used to delimit and direct the initial search results, from which we established the following research questions: (1) What do authors of current research say about human application? (2) What necessary technical improvements are identified in the research? (3) On which issues do the authors agree? and (4) What future research priorities emerge in the studies? We used the Cohen kappa statistic to validate the interrater reliability.

**Results:**

The 13 articles included in the review demonstrated the feasibility of cartilage bioprinting in live animal studies. Some investigators are already considering short-term human experimentation, although technical limitations still need to be resolved. Both the use and manufacturing process of stem cells need to be standardized, and a consensus is needed regarding the composition of hydrogels. Using on-site printing strategies and predesigned implants may allow techniques to adapt to multiple situations. In addition, the predictive capacity of implant behavior may lead to optimal results.

**Conclusions:**

Cartilage bioprinting for surgical applications is nearing its initial use in humans. Current research suggests that surgeons will soon be able to replace damaged tissue with bioprinted material.

## Introduction

Cartilage is a specialized connective tissue devoid of nerves, blood, and lymph vessels. It has flexible characteristics and consists of an abundant extracellular matrix and chondrocytes. Articular cartilage lesions do not heal spontaneously and lead to impaired function, progressive disability, and decreased quality of life [1]. Traumatic and degenerative cartilage injuries represent one of the most challenging and frustrating clinical scenarios.

Medical specialties have a long history of adopting new solutions for patient problems, including new techniques to repair or replace damaged tissue, such as total joint replacement by orthopedic surgeons, cornea replacement in ophthalmology, and repairing malformations or congenital absence of the ear (ie, microtia) [2]. Repairing or replacing damaged or absent cartilage structures, such as the ear or nose, presents a significant challenge in reconstructive plastic surgery; in these cases, a clinically conceivable procedure needs to be created, because current procedures often involve multiple surgeries [3] and complications, such as infections, tissue necrosis, pain, and the risk of an undesirable result [4].

Bioprinting technology (ie, three-dimensional [3D]) is a new approach that allows the regeneration of cartilaginous structures using cartilaginous cells in a biocompatible environment. The 3D shape of the bioprinting product can be exact, which is very important in nasal septum or external ear reconstruction [5].

Tissue engineering and regenerative medicine are life science fields that use the principles of tissue engineering to regenerate damaged structures or create new ones [6]. Better understanding of how to optimize patient care can improve outcomes and quality of life, allowing more efficient use of health resources. Results of previous research [7,8] suggest that the most logical next step is to examine surgeons’ responses to this new therapeutic possibility. Reviewing, analyzing, and categorizing the different research activities in this new field [9] will help define the scope and depth of future research and identify gaps in critical knowledge [10]. This review synthesizes published studies on bioprinted cartilage to accomplish the following: (1) identify the current state of cartilage bioprinting, (2) identify the technical issues associated with human application, and (3) highlight the need to extend the advanced knowledge to clinicians.

## Methods

### Overview

Previous literature in this field lacks specificity; therefore, a scoping study methodology was chosen to correctly identify information gaps and precisely illustrate future research needs. The scoping review system creates a map of the published literature to explore the methodological and empirical differences in various knowledge areas.

### Study Design

#### Overview

A scoping review methodology was chosen because it is more exploratory and less methodological than systematic reviews; this was essential to meet the study objectives. The research strategy was modified according to Arksey and O'Malley’s [11] methodological framework, which proposes a five-stage transparent process for replicating research strategies to increase the reliability of the results. The first stage clarifies and links the study purpose and the research questions; stage two balances feasibility with the breadth of the research process; stage three includes study selection; stage four involves mapping the data; and stage five summarizes the findings.

#### Clarifying and Linking the Purpose to Research Questions

This study aimed to identify the current status of cartilage bioprinting and the associated influence on clinical use, as well as to subsequently improve the information that reaches surgeons. The following research questions guided the search:

1. What do authors of current research say about human application?

2. What necessary technical improvements are identified in the research?

3. On which issues do authors agree?

4. What future research priorities emerge in the studies?

**Figure 1:**
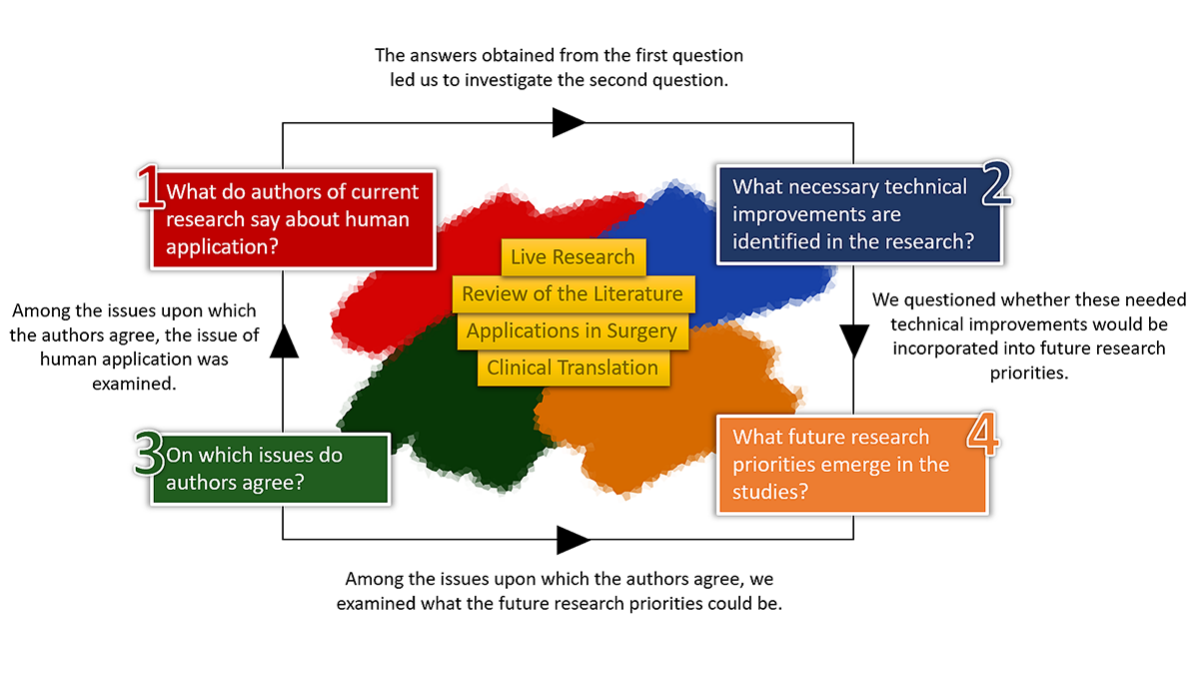
Conceptual framework of the scoping review.

After determining the research questions, we developed a conceptual framework to define and map the key concepts of bioprinted cartilage and to identify research gaps that may hinder using bioprinting techniques in human applications (see Figure 1). The conceptual framework guided both the analysis and the systematic presentation of the summarized data. The four research questions comprised the main branches of the framework, and the extracted data were categorized into four blocks, which answer our research questions.

#### Balancing Viability With the Breadth of the Process

The bibliographic search was conducted between January and March 2019 and included Scopus, Web of Science, and PubMed databases. Choosing the correct key terms was critical to facilitating maximum coverage of the related research literature [12]. We used Medical Subject Headings (MeSH) terminology to increase search sensitivity: “bioprinting” AND “surgery” AND “cartilage” OR “surgical procedures.” We also examined each article’s reference list and conducted additional Google Scholar searches on research terms available in the gray literature. This expanded the search by adding the following terms: #bioprinting, #articular cartilage, #tissue engineering, #cartilage, #stem cells, #scaffolding, #biofabrication, #cartilage regeneration, #surgery, #transplantation, #cartilage tissue engineering, and #clinical translation.

#### Study Selection

**Figure 2:**
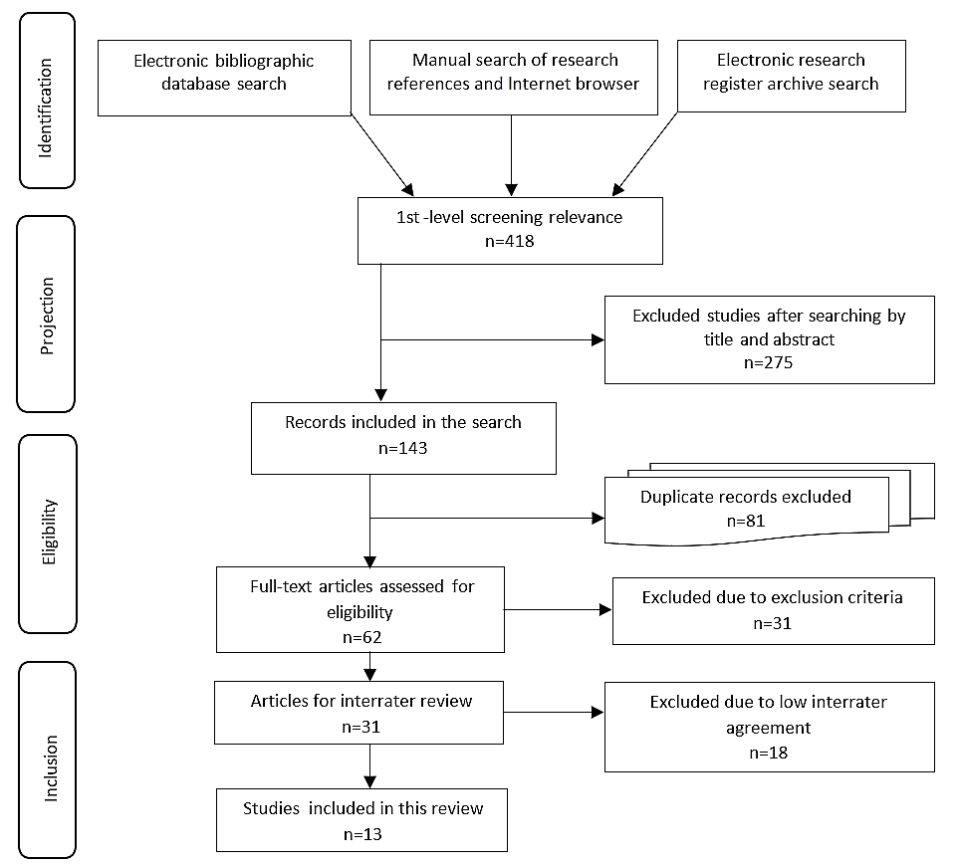
Preferred Reporting Items for Systematic Reviews and Meta-Analyses (PRISMA) flow diagram for the scoping review process.

Bioprinted cartilage technology has changed in recent years; consequently, only a limited number of articles, some of which were already in the authors' bibliography archives, were included. Scoping reviews [13] are used to map underlying concepts; therefore, as in other types of knowledge synthesis [14], it is essential to define the methods. In 2015, the Joanna Briggs Institute published the methodological guidelines [15] for presenting a broad view of the evidence, regardless of study quality; clarifying key concepts; and identifying gaps [16]. This methodology involves incorporating a checklist to increase method transparency, judge validity and reliability, and adequately handle the search [17]. Among the existing forms of presentation, we focused on the revised and expanded Preferred Reporting Items for Systematic Reviews and Meta-Analyses-Rapid Reviews (PRISMA-RR) [18]. Figure 2 illustrates the transparency of the article selection.

The electronic database search, the Internet hand search, and the archive database search identified 418 articles; 275 were excluded because the main concepts of our search were only cited in the context of this work. A total of 81 duplicates were also excluded as well as 31 articles due to exclusion criteria (see Table 1). Interrater agreement was analyzed for the remaining 31 articles using the Cohen kappa statistic [19-21], which indicated a moderate level of agreement among our evaluators and yielded a total of 13 articles for analysis.

#### Extracting and Charting the Results

Kok and Schuit [22] proposed a method to map research contributions to improve the impact of research on the population’s health. The method focuses on producing anticipatory processes and extending, disseminating, and using knowledge. The articles selected for analysis through evaluator agreement were all published between 2016 and 2019.

The collected articles were organized by author, title, year, country, and type of article (see Table 2). The selected articles originated from the United States (4/13, 31%) [23-26], China (2/13, 15%) [27,28], Korea (2/13, 15%) [29,30], Sweden (2/13, 15%) [31,32], Australia (2/13, 15%) [33,34], and Canada (1/13, 8%) [35].

#### Reporting the Findings

The articles were classified by following types of study design:

1. Live research (ie, carried out on animals).
2. Literature reviews.
3. Surgical applications.
4. Clinical translation (ie, a review methodology focused on clinical application).

We also referenced the summarized information of each article for future interpretations.

#### Availability of Data and Materials

The data used and analyzed in this study are available from the primary author upon reasonable request.

**Table 1:** Inclusion and exclusion criteria.

Criteria	Inclusion criteria	Exclusion criteria
Language	English	Non-English
Year of publication	2009-2019	Before 2009
Peer reviewed	Yes	No
Study design	Original research In vivo study Literature revision Description of surgical procedures	Clinical trials in phase I/II Studies conducted in the laboratory environment
Ethical permission	Obtained	Not obtained

**Table 2:** Selected articles.

Authors	Title	Year	Country	Type of article
Di Bella et al [34]	In situ handheld three-dimensional bioprinting for cartilage regeneration	2018	Australia	Live research
Onofrillo et al [33]	Biofabrication of human articular cartilage: A path towards the development of a clinical treatment	2018	Australia	Live research
You et al [35]	Homogeneous hydroxyapatite/alginate composite hydrogel promotes calcified cartilage matrix deposition with potential for three-dimensional bioprinting	2019	Canada	Live research
Apelgren et al [31]	Skin grafting on 3D bioprinted cartilage constructs in vivo	2018	Sweden	Live research
Wu et al [25]	Three-dimensional bioprinting of articular cartilage: A systematic review	2018	United States	Literature review
Dhawan et al [26]	Three-dimensional bioprinting for bone and cartilage restoration in orthopaedic surgery	2019	United States	Literature review
Leberfinger et al [24]	Concise review: Bioprinting of stem cells for transplantable tissue fabrication	2017	United States	Literature review
Apelgren et al [30]	Chondrocytes and stem cells in 3D-bioprinted structures create human cartilage in vivo	2017	Korea	Surgical application
Yi et al [29]	Three-dimensional printing of a patient- specific engineered nasal cartilage for augmentative rhinoplasty	2019	Korea	Surgical application
Kaye [32]	A 3-dimensional bioprinted tracheal segment implant pilot study: Rabbit tracheal resection with graft implantation	2019	Sweden	Surgical application
Li [27]	In situ repair of bone and cartilage defects using 3D scanning and 3D printing	2017	China	Surgical application
Boushell [23]	Current strategies for integrative cartilage repair	2017	United States	Surgical application
Liu et al [28]	Recent progress in cartilage tissue engineering: Our experience and future directions	2017	China	Clinical translation

## Results

### Overview

Table 2 lists the articles included in this review. All reviewed studies contributed to understanding the complexity of applying cartilage bioprinting in humans. Table 3 summarizes the authors’ contributions regarding the first research question. This grouping allowed us to identify the approach according to the lines of research. The articles included in the group of in vivo studies emphasized the applied aspects of technology development, such as the elements that constituted the process (eg, bioink and its composition, replicability and cell viability, and the bioprinter), as well as bioprinting intervention strategies that included the use of a Biopen (ie, a manual bioprinter) with preclinical results in large animals. This is the strategy closest to human experimentation, according to the authors who used it.

### Clinical Translation

Questions that arise from the studies cover a wide range of possibilities. Key elements for clinical translation included scalability and lesion characteristics, such as different lesion geometries and measurements. Insights from surgical application studies included problems specific to orthopedic, plastic, and otorhinolaryngology surgery. To progress toward human application, each surgical strategy must overcome these application-specific challenges. In addition, Boushell et al [23] opened debate on the scaffold versus cellular approaches. Li et al’s [27] translational study provided specific reading aimed at clinical professionals to establish synergies with basic research. Its goal was to reach surgical professionals not directly involved in the research.

Table 4 details the technical improvements identified in the studies that were necessary to continue progressing toward human application. In general, they involve two concepts: cellular sources and biomaterials, including scaffolds and hydrogels. Onofrillo [33], Apelgren et al [31], and Leberfinger [24] prioritized the need to develop protocols for obtaining cells; they also recognized that, despite variable sources, all cells must maintain chondrogenic capacity, not cause morbidity at the donor site, expand easily in the culture without losing phenotype, and support the mechanical load in the joint case. Di Bella et al [34], You et al [35], and Wu et al [25] presented disparate technical aspects that should be improved, since they followed different research paths. However, they all identified necessary biomaterial and scaffolding improvements, although the types of recommended improvements did not coincide.

**Table 3:** Authors’ perspectives about current research for human application.

Type of article, authors, year		Details and authors’ perspectives
**Live research**		
	Di Bella et al, 2018 [34]	Authors used the Biopen manual printing system in the operating room for implanting cartilage directly into the bed of a lesion. They suggested that this system would improve the possibility of use in humans because it facilitates in situ implant creation, and they have demonstrated clinical efficacy and safety in large animals. They have not detected intraoperative or perioperative complications. The preliminary data obtained on the safety and stability of the in vivo characteristics of the implanted cartilage suggest that use of the technique in humans may not be far off.
	Onofrillo et al, 2018 [33]	Authors used the Biopen system and contributed to defining a possible clinical bioprinting protocol for application in humans. They studied the cell viability and characteristics of bioinks to ensure that the created cartilaginous tissue was similar to native tissue. They concluded that their discoveries allow customized repair of cartilaginous lesions in humans.
	You et al, 2019 [35]	Authors studied hydrogel composition to improve the printing and dispersion of particles in situ. They reported that their investigations represent a step toward implantation in humans because they improved the mimesis with the osteochondral junction.
	Apelgren et al, 2018 [31]	Authors in this mouse study investigated implanting skin-coated chondrocytes for application in auricular reconstructive surgeries. They suggested that for human surgery applications, it is necessary to show that the reconstruction procedure is clinically conceivable and replicable. The results increased the clinical potential in humans.
**Literature reviews**		
	Wu et al, 2018 [25]	Authors demonstrated that articular cartilage bioprinting is a tissue engineering strategy that has potential translational value.
	Dhawan et al, 2019 [26]	Authors reported scalability, geometric, and lesion measurement problems that may pose barriers to human application. They suggested the use of tomographic images for the design of the implant and its accuracy.
	Leberfinger et al, 2017 [24]	Authors analyzed the essential elements of the bioprinting process, the different cellular sources, the bioink, and the implants with and without scaffolding. They conducted a cost-effectiveness study to evaluate the feasibility of clinical translation.
**Surgical applications**		
	Apelgren et al, 2017 [30]	Authors studied the creation of cartilage with human chondrocytes in vivo; they also quantified the chondrogenic potential in combination with mesenchymal stem cells in bioprinting constructions implanted in mice for their application in plastic surgery.
	Yi et al, 2019 [29]	Authors established a procedure based on a 3D computer-aided model to generate a customized nasal implant design. They reported that computer design is necessary for creating human implants.
	Kaye et al, 2019 [32]	In their pilot study, authors investigated the feasibility of introducing a functional in vivo tracheal replacement in rabbits.
	Li et al, 2017 [27]	Authors conducted a study to improve the cartilage defect imaging in orthopedic surgery for implementation in humans; they concluded that it is necessary to optimize the imaging process.
	Boushell et al, 2016 [23]	Authors defended the use of scaffolding versus the cellular approach in human applications for orthopedic surgery. They noted that scaffolding requires fewer chondrocytes and that the functional mechanical properties of the tissue are more easily achieved by scaffolding.
**Clinical translation**		
	Liu et al, 2017 [28]	Authors identified key aspects in clinical translation for human use in orthopedic surgery: (1) integration with subchondral bone for correct load distribution, (2) ensure the coincidence in the mechanical properties between the native cartilage and the implant to avoid the degradation caused by the tensional disparity, (3) guarantee resistance under deformations and movements, and (4) recapitulate different zonal architecture to achieve the structure-function relationship of the native cartilage.

**Table 4:** Needed improvements in the technical aspects.

Type of article, authors, year		Needed improvements
**Live research**		
	Di Bella et al, 2018 [34]	The chemical characteristics of the biomaterial need to be improved to ensure adhesion of the implant in depth and thickness at the site of the injury.
	Onofrillo et al, 2018 [33]	The ideal cell type for cartilage regeneration is still a matter of debate, as it has to be ensured that the cells obtained have a proven chondrogenic capacity, do not cause morbidity in the donor site, and are easily expandable in the culture without losing their phenotype.
	You et al, 2019 [35]	The properties of hydrogels must be improved. The authors’ findings show the promise of alginate/hydroxyapatite hydrogel printed on 3D scaffolds with a porous structure for calcified and bioprinted cartilage formation.
	Apelgren et al, 2018 [31]	A model of bioprinted cartilage for an atrium has the potential to have a very elaborate form; however, authors state that they still need to obtain an adequate skin coverage that allows for highlighting of these high-resolution forms in the in vivo application.
**Literature reviews**		
	Wu et al, 2018 [25]	The mechanical strength in bioprinting without scaffolding should be further investigated, along with the toxicity in the implanted cells. For the authors, bioprinting without scaffolding offers many possibilities for the future since it reaches a relatively high initial cell density without the inclusion of biomaterials; this translates into more space for extracellular matrix deposition as well as facilitating better cell-cell interaction, generating biomimetics, preserving cellular functionality, and eliminating tissue biodegradation.
	Dhawan et al, 2019 [26]	The protocolization of technological manufacturing strategies of bioprinted cartilage needs improvement in order to allow its scalability. This technology would have the ability to manufacture tissues in clinically relevant volumes and address defects of different sizes and geometries.
	Leberfinger et al, 2017 [24]	To ensure the clinical safety of obtained cells, necessary manufacturing facilities must be created for processing, including isolation facilities in hospitals, to facilitate the transition toward clinical use. Among the cellular sources that could be used, the authors point out embryonic stem cells, induced pluripotent cells, and adult stem cells from bone marrow and adipose tissue.
**Surgical applications**		
	Apelgren et al, 2017 [30]	A significant challenge in reconstructive plastic surgery is the approach that allows the regeneration of cartilage structures using autologous cells dispersed in biocompatible scaffolds. Several problems associated with this method have not yet been investigated or resolved, including load-bearing capacities, shear strength, elastic characteristics, and resistance to degeneration.
	Yi et al, 2019 [29]	In patients who require a nasal implant, the postoperative characteristics of the skin that will cover the implant must be improved to ensure that it is not affected by the external pressure generated later, nor by the degradation of the biomaterials in the long term. The authors propose a pre- and postoperative control algorithm that calculates all the variables.
	Kaye et al, 2019 [32]	In their in vivo study of the implantation of a trachea seeded with cells, authors detected the need to cover the implant with a membrane to avoid inflammatory reactions and stenosis of the light when applied in humans. These findings are essential for the future of reconstruction and implantation of tracheal grafts.
	Li et al, 2017 [27]	It is necessary to visualize the cartilaginous defect with computed tomography, synthesize suitable biomaterials, and print hydrogels in a personalized way in a short time. The use of a specific bioprinter to carry out this process is necessary to achieve the objective of personalized implants in situ through bioprinting.
	Boushell et al, 2016 [23]	The characterization, optimization, and standardization of models in large animals will be critical for the next phase of the investigation for cartilage repair. In addition to the development of better culture models, more research is needed to fully understand the long-term maintenance and homeostasis interface of the integration strategies to ensure the success of the procedure.
**Clinical translation**		
	Liu et al, 2017 [28]	Improving collaboration between materials scientists and experts from other fields related to tissue engineering is of vital importance to obtain hydrogels with balanced mechanical properties, electrical conductivity, degradation rate, biocompatibility, and chondro-inducing properties.

The surgical application studies focused on certain surgical approaches and identified specific technical improvements needed to obtain better results; improvements included an algorithm to ensure that the nasal implant is not degraded or subjected to excessive long-term pressure [29]; a process to guarantee the characteristics of the skin of the ear in plastic surgery [31]; and in otorhinolaryngology, the use of a membrane trachea coating and image processing to optimize surgical results [36].

Table 5 reflects those aspects that were identified as recurrent among the different groups. An elaborate synthesis of the elements shared across the studies was completed. Analyzing these recurring elements allowed us to understand the group positions and identify the main shared aspects.

**Table 5:** Issues upon which the authors agreed.

Type of article, authors, year		Issues
**Live research**		
	Di Bella et al, 2018 [34]	Once tested on animals, it is necessary to design a strategy to detect whether the implant has been kept in situ in order to move on to human trials.
	Onofrillo et al, 2018 [33]	To guarantee stability in situ, a gradient of osteogenic and chondrogenic growth factors should be added to the hydrogel to promote selective tissue differentiation that would allow the formation of bone and cartilage, acquiring the complete osteochondral unit.
	You et al, 2019 [35]	To ensure that the skin regeneration characteristics obtained in mice can be extrapolated to humans, they will need to perform the same experiment on large animals with regenerative capacities more similar to humans.
	Apelgren et al, 2018 [31]	To ensure cell viability and optimal measurement of the implant to preserve atrial features, large animals must be investigated.
	All authors	Common opinion: the next steps are to expand the research on large animals and to prolong the monitoring time to confirm the preliminary results of cell viability, in situ conservation of the implanted tissue, maintenance of the mechanical characteristics, and long-term lateral integration.
**Literature reviews**		
	Wu et al, 2018 [25]	Advances so far have allowed the replication of the anatomical structures, the biological function, and the mechanical properties of the implant; however, it is necessary to continue analyzing the viability and the autoimmune response of the implanted cells before the various stimuli to which they are subjected in the living organism are activated.
	Dhawan et al, 2019 [26]	These specific designs for the patient must be protocolized, not only concerning the geometry of the implant but also at the anatomical level of defect.
	Leberfinger et al, 2017 [24]	The authors have found that the lack of standardized and efficient differentiation protocols of stem cells leads to variable results among groups of researchers.
	All authors	Common opinion: the authors consider it essential to protocolize cell differentiation and to ensure viability, chondrogenic differentiation, scalability, and control of autoimmune reactions for implantation in humans.
**Surgical applications**		
	Apelgren et al, 2017 [30]	It is necessary to extend the control period in experimental animals to assess stability and long-term integration in order to ensure the absence of malignancy.
	Yi et al, 2019 [29]	It is necessary to ensure the long-term maintenance of the implant shape in large constructions and to control the central hypoxia of the implant; this would avoid an insufficient supply of oxygen and nutrients to the cells through the hydrogel that allows its diffusion.
	Kaye et al, 2019 [32]	When an ideal tracheal replacement graft is constructed, the ability to fully integrate in vivo depends on its immunogenicity and its ability to promote revascularization. Also, any tracheal replacement graft must be a mechanical and functional complement similar to the native trachea.
	Li et al, 2017 [27]	It is necessary to establish a methodology for in situ printing mediated by images for personalized implants. The proportion of balanced hydrogel between the speed of printing and the maintenance of cell viability must be considered as an indispensable part of the bioprinting. The structural characteristics and zonal organization of normal articular cartilage should be considered.
	Boushell et al, 2016 [23]	Further exploration of appropriate culture models is required to obtain tissue integrity and prevent ectopic calcifications. A long-term solution for the treatment of full-thickness cartilage defects must be developed.
	All authors	Common opinion: long-term evaluation is essential to ensure the maintenance of the form, the mechanical and functional resistance of the implant, as well as vascularization. A clinically relevant methodology must be established.

Table 6 reflects the lines that suggest prioritizing diverse groups. These were derived from the specific research studies, and therefore there was no shared opinion. At a general level, however, more research on manufacturing strategies to establish the role of scaffolding and accelerate integration of native and newly formed cartilage is required. Finally, when the technology is available to humans, the results obtained from bioprinted cartilage should be compared to the traditional gold standard.

**Table 6:** Future research priorities proposed by the authors.

Type of article, authors, year		Future research priorities
**Live research**		
	Di Bella et al, 2018 [34]	Authors propose the use of the Biopen for its ease of use, which does not require prior training by the surgeon. They highlight the need for more trials to evaluate the biomechanical characteristics of cartilage.
	Onofrillo et al, 2018 [33]	Authors propose studying the phenotype, cell migration, matrix deposition, proteolytic activity, and the rate of degradation of the construct; they propose this in order to evaluate and correlate with the formation of new cartilage and to better understand the interaction between human adipose-derived stem cells and the gelatin methacrylate/hyaluronic methacrylate cross-linked hydrogel.
	You et al, 2019 [35]	Authors propose that the combination of alginate/hydroxyapatite should be considered a critical component for the regeneration of the osteochondral interface of the skeletal joints.
	Apelgren et al, 2018 [31]	Authors propose a methodology capable of evaluating the in vivo proliferation of chondrocytes, alone and in a combination with mesenchymal cells. They suggest that their technique is viable in that it maintains the proliferative capacity of cartilage over time.
**Literature reviews**		
	Wu et al, 2018 [25]	Authors propose studying the mechanical forces that are applied to the knee and that a semiconfined compression is a good way to mimic the native mechanical environment in future studies. Thus, it will be possible to study how the mechanical stimuli regulate the cell activities in the bioprinted constructs.
	Dhawan et al, 2019 [26]	Authors propose that when the technology is available for humans and once bioprinted cartilage implants are obtained, they can be compared with the traditional gold standard.
	Leberfinger et al, 2017 [24]	Authors recommend carefully studying the growth factors that are part of the biomaterials before proceeding to standardize the bioprinting and implantation of the graft.
**Surgical applications**		
	Apelgren et al, 2017 [30]	Authors propose exploring other cellular sources from in vivo studies to compare before standardizing the bioprinting process. Stem cells derived from adipose tissue that could support chondrogenesis are proposed. They propose lengthening the study time in vivo to confirm the stability of the cartilage shape, elastic characteristics, and integrity.
	Yi et al, 2019 [29]	Authors propose investigating algorithms for implant applications in other types of tissues, since they argue for the high versatility of the technology; the availability of various extracellular matrix materials of the tissue; and the pluripotency of the human adipose-derived stem cells. This contributes to the structural accuracy of the nasal cartilage, and the hydrogel provides a favorable environment for chondrogenic differentiation and the formation of neocartilage.
	Kaye et al, 2019 [32]	Authors propose that for uses of cartilage bioprinting where the implant should not be integrated (eg, the trachea), the study of separating membranes that allow the implanted organ to remain isolated is recommended.
	Li et al, 2017 [27]	Authors propose the use of bioprinting with assistance from a scanned image for significant segmental defects of long bones and open chondral lesions. The technique allows a dual approach to cartilage and bone.
	Boushell et al, 2016 [23]	Authors propose new strategies of clinical management based on research with personalized scaffolds combined with chemotactic factors; these would give rise to a stable, functional repair with good long-term results.
**Clinical translation**		
	Liu et al, 2017 [28]	Authors propose elaboration of a guide of Good Manufacturing Practice that allows the complete production of cartilaginous grafts on a large scale. Most of the new developments in engineering of cartilage tissue have not yet translated into measurable improvements for clinicians.

## Discussion

### Principal Findings

In recent years, there has been an increase in the annual publication of articles on cartilage bioprinting, contributing to the knowledge and management of this process. The methodology adopted in this review allowed us to analyze 13 articles and present systematically summarized data. No clinical trials in humans have been identified to date. Tests with large animals presented some challenges and suggested possible strategies [37]. In this context, the reviewed articles provided polyhedral visions to the problem and proposed lines of research to progress toward human application. Identifying four groups based on research characteristics allowed us to establish synergies, understand confluences across studies, and highlight specific problems that surfaced as well as potential problems that may emerge as the field advances.

The Biopen [33,34] is the technique most likely to be applied in humans in the short term. The Biopen arose out of a collaboration between researchers at the University of Wollongong-based Australian Research Council Centre of Excellence for Electromaterials Science and orthopedic surgeons at St. Vincent’s Hospital in Melbourne. The Biopen technique is based on a small bioprinter that is easy to handle and is loaded with biological inks composed of stem cells inside a biopolymer, which in turn is protected by a second layer of hydrogel. The exchange of injectors allows different cells to be deposited at different concentrations on the surface to be repaired and, thus, recreates the zonal anatomy of native cartilage. It is then solidified by an ultraviolet light embedded in the pen. It is an attractive proposition for surgeons since its use does not require a long learning curve. The Biopen allows precise positioning of cells and biomaterials, rapid placement at the defect site, and minimal manipulation by the surgeon. Other authors advocate the predesign rather than in situ design of the implant: Yi et al [29], Apelgren et al [31], Li et al [36], Kaye et al [32], and Boushell et al [23]. These five studies focus on surgical applications in plastic surgery, otorhinolaryngology, and orthopedics.

The characteristics of these approaches make it difficult to use on-site technologies, while the preoperative design of the implants is necessary. In Kaye et al’s study [32], tracheal substitution started from a decellularized extracellular matrix trachea and subsequently seeded cells. Currently, in situ application of the technique appears to be restricted to joint injuries, despite being in a more advanced state of research. The image-mediated design, with algorithms such as those proposed by Yi et al [29], allows an implant, as similar as possible, while allowing for preoperative assessment of pressure and skin growth effects in plastic surgery implants. For Li et al [36] and Yi et al [29], the use of images is a line that must be exploited to ensure functional transplants with preservation capacity in both nasal and orthopedic applications. The Biopen technique would make it possible to ignore image studies, which contain a certain margin of error. This is evident in both Li et al’s [36] and Yi et al’s [29] studies, where they recommended technical improvements for obtaining and processing previous images to guarantee the implant design and facilitate optimal implantation.

Both impression approaches face a series of challenges, including maintenance of the implant form, cell viability, and mechanical resistance. The interface between the implant and adjoining native tissues also needs to be addressed. Form maintenance encompasses different strategies, such as the use of desacralized structures, as described by Kaye et al [32] with tracheal implants.

Scaffolds can contribute rigidity and mechanical resistance to the implant. In scaffolding, a structure with synthetic biopolymers provides mechanical support to maintain shape and load, while hydrogel provides a biological environment for regeneration of bioprinted cartilage [5]. Boushell et al [23] advocated the use of scaffolds insofar as they require a lower cellular concentration and facilitate the mechanical properties of the implant, which seems to adopt better mechanical-functional behaviors. On the other hand, Biopen techniques do not require a classic scaffold; however, they should guarantee both peri- and postoperative safety, functionality, and nondegradation of the construct, while scaffolds must ensure lateral integration of the implant. There is debate about the usefulness of scaffolds in orthopedic surgery. To date, lateral integration of the implant has not been confirmed with enough clarity.

Implant integration and fixation are aspects that can affect all the analyzed proposals. Correct integration and fixation of the neocartilage requires geometric measures of the osteochondral lesion’s total volume. Resistance to implanted cartilage degradation should be guaranteed in the long term, whether or not scaffolding is used. Wu et al [25] proposed semiconfined compression as an excellent way to mimic the native mechanical environment in future studies, facilitating research on how mechanical stimuli regulate cell activities in bioprinted constructs.

The risk of inflammation or the contraction or deformation of the implanted tissue, either with or without a scaffold, affecting the end result should not be overlooked. Kaye et al’s [32] work highlighted this difficulty in tracheal surgery; Yi et al [29] advocated greater precision in the algorithm to ensure that there is no modification of the postoperative nasal implant related to external causes. Postoperative cellular viability, such as maintaining cellular replication over time, must be analyzed by methods such as those proposed by Apelgren et al [30]. To date, the Biopen technique has not provided long-term viability results in large animals.

The hydrogels used must respond to a variety of biological needs, ensuring balanced mechanical properties, electrical conductivity, degradation rate, biocompatibility, and chondro-inducing properties [27]. Specific equilibria can be found in the speed of printing and the maintenance of cell viability [36]. In plastic surgery and otorhinolaryngology, they must also allow the correct irrigation of the tissue to avoid situations of hypoxia. The combination of biomaterials, such as alginate/hydroxyapatite [35] or a cross-linked gelatin methacrylate/hyaluronic methacrylate [33], should be considered a critical component for regeneration of the osteochondral interface in orthopedic surgery.

The cellular source of the implants is the last element of debate. There are two issues: cellular origin and cell treatment. The cells can be obtained from adult tissue-derived stem cells (ie, fat cells, bone marrow, and others), mesenchymal stem cells, autologous chondrocytes, and induced pluripotent cells. Researchers used two types of cells in the reviewed works: mesenchymal stem cells and stem cells derived from adipocytes. Cells extracted from the patient encounter extraction problems, but the chondrogenic capacity facilitates cellular processing and regulatory requirements, which are much higher with stem cells. Standardizing all steps in the process (eg, cell differentiation, the composition of the hydrogels, and the speed of printing) is necessary to enable translation to humans [27].

### Identified Gaps

Although approaches differ depending on the study type and the application, a series of gaps and challenges were identified that were shared across studies, although there are differences in the ease of resolution, functional technique, and surgical strategies:

1. Optimum integration with the host subchondral bone and cartilage must be achieved.
2. The biological, biomechanical, and degradation properties of the bioprinted cartilage must be ensured.
3. A systematic manufacturing process must be developed and implant preservation, cellular sources, and the role of scaffolds must be optimized.
4. Clinical safety related to the effects of implants on native tissues must be examined.

### Challenges

#### Overview

Surgical challenges similar to allogeneic organ transplants, including cellular ischemia and size adjustment, will persist. According to Li et al [36], the size match can be planned before surgery with computed tomography and computer-aided design images [38]. The implanted tissue must be composed of biocompatible materials that are integrated into the native cells, allowing growth and preventing an immune response. Ethical dilemmas and regulatory problems are also likely to arise as this technology advances.

#### Ethical Dilemmas

To avoid an immune response in current transplants and lifelong treatment, adult stem cells offer the ability to produce autologous tissue that prevents the need for immunosuppressive therapy [24]. Support and biocompatible biological components must have a low inflammatory response to prevent the appearance of macrophages [39]. Even small changes in the chemical, physical (ie, structure and degradation), and mechanical properties of bioprinting materials can affect the integrity and biocompatibility of the structural component and, ultimately, the performance after it is implanted [40].

Two crucial nonclinical challenges will also affect implementation of this technology: regulation and costs. The reviewed studies focused primarily on specific technical aspects, except for the Leberfinger et al study [24], which investigated cost relationships. In addition, Liu et al [27] suggested a need for useful practice manuals to facilitate both the translation and regulation of the techniques.

#### Regulation

Currently, when cells are modified and combined with a scaffold that provides physical support for the growth of new tissue, they are regulated as biological products in the United States and as advanced therapy drugs in the European Union (EU) [41]. The regulatory aspects that align development of these combined products lack clarity, both in the EU and in the United States. There is also uncertainty regarding the potential impact of current proposals to amend the EU directive on medical products [42].

#### Costs

One concern associated with personalized regenerative medicine is the uncertainty regarding the cost of obtained tissues [40]. Costs associated with cell acquisition and processing, scaffold manufacturing, bioreactor maturation, surgical implantation, and postoperative care are also likely to be substantial, but it is not clear how they will compare with the current cost of transplants [43]. As with any new scientific advance, costs will probably decrease as technology evolves and becomes more efficient.

#### Implications for Future Research

Bioprinting technologies are unique in that they allow a certain pattern of multiple cell types and materials to recreate the native structure of cartilage [44]. Future studies should evaluate other sources of multipotent stem cells, such as stem cells derived from adipose tissue or from mesenchymal or other cells, to support chondrogenesis. These stem cells can be easy to collect, and some studies report that they have substantial proliferative potential [30].

Collectively, the analyzed studies demonstrate the feasibility of cartilage engineering and underscore the need for a continuous biological barrier between the neo-cartilage and the bone region. It is likely that the biphasic design alone is not sufficient to achieve consistent and functional cartilage, as well as formation and integration into the subchondral bone [30]. Peripheral distribution in the matrix formation, as well as correct orientation of the collagen fibers and mechanical resistance to tension, are vital elements in cartilage tissue engineering [33]. Although in vivo testing has been conducted in large animals, before progressing to human trials it is necessary to specify and resolve the detected gaps to establish the necessary physical and biomechanical characteristics, address potential implant degradation, and ensure transverse integration of the graft in the host.

### Strengths and Limitations

One limitation is the heterogeneity of the selected articles. Evaluating the methodological quality of the included studies was not within the scope of this review, which aimed to identify and synthesize the key concepts in cartilage bioprinting research. There may be additional relevant works that were not identified by the search strategy used in this review.

This concise review presented the evolving technology of cartilage bioprinting and its main components, with a particular focus on clinical translation. This work contributes a summary and update of current research in this area, which can be made available to clinicians to facilitate a better understanding of this new technology.

### Conclusions

Human applications for bioprinted cartilage are likely to emerge in the near future. Advanced research on bioprinted cartilage can become a spearhead for adapting the technology to bioprint other types of tissue. On-site printing strategies and predesigned models can adapt to different situations. In addition, as imaging technology advances, processing implants and identifying the predictive capacity of implant behavior will allow better results. Regulation of the technology across different countries and cost-effectiveness of the technique will also need to be addressed in future studies.

## Abbreviations

3D: three-dimensional
EU: European Union
MeSH: Medical Subject Headings
PRISMA-RR: Preferred Reporting Items for Systematic Reviews and Meta-Analyses-Rapid Reviews
